# Effects of long and short ejaculatory abstinence on sperm parameters: a meta-analysis of randomized-controlled trials

**DOI:** 10.3389/fendo.2024.1373426

**Published:** 2024-05-17

**Authors:** Arturo Lo Giudice, Maria Giovanna Asmundo, Sebastiano Cimino, Andrea Cocci, Marco Falcone, Marco Capece, Ali Saber Abdelhameed, Paolo Capogrosso, Afonso Morgado, Georgios Tsampoukas, Celeste Manfredi, Giorgio Ivan Russo

**Affiliations:** ^1^ Urology Section, University of Catania, Catania, Italy; ^2^ Urology Section, University of Florence, Florence, Italy; ^3^ Urology Section, University of Turin, Turin, Italy; ^4^ Urology Section, University of Naples Federico II, Naples, Italy; ^5^ Department of Pharmaceutical Chemistry, College of Pharmacy, King Saud University, Riyadh, Saudi Arabia; ^6^ Urology Section, University of Varese, Varese, Italy; ^7^ Urology Section, University of Porto, Porto, Portugal; ^8^ Urology Section, The Great Western Hospital, Swindon, United Kingdom; ^9^ Department of Urology, University of Catania, Catania, Italy

**Keywords:** semen parameters, sperm parameters, ejaculatory abstinence, sexual abstinence, DNA fragmentation

## Abstract

**Purpose:**

This study aimed to investigate the effects of ejaculatory abstinence on sperm parameters.

**Methods:**

This analysis was registered in PROSPERO (CRD42023472124). We performed a search on PubMed using the following text terms: ((“sperm parameters” OR “sperm analysis” [Mesh]) AND (“sperm DNA fragmentation” OR “DNA fragmentation” [Mesh]) AND (“sexual abstinence” [Mesh] OR “abstinence”)) and an advanced search in Scopus using the terms (“sperm parameters” OR “sperm parameters” OR “DNA fragmentation”) AND (“abstinence”). The sperm parameters that were investigated were sperm volume, total sperm motility, progressive sperm motility, sperm concentration, sperm morphology, and sperm DNA fragmentation (SDF). A two-day cut-off as a “short” or “long” abstinence period has been defined.

**Results:**

Thirteen studies published between 2013 and 2022 were included in this meta-analysis. A total of 2,315 patients, ranging from 6 to 836 from each cohort, were enrolled in the study. We showed that longer abstinence time was associated with greater sperm concentration (mean difference [MD]: 8.19; p <0.01), sperm volume (MD: 0.96; p <0.01), and higher SDF (MD: 3.46; p <0.01), but lower progressive sperm motility (MD: −1.83; p <0.01). Otherwise, no statistically significant difference was observed in patients with longer vs. shorter abstinence times regarding total sperm motility (MD: −1.83; p = 0.06). Meta-regression analysis showed that days of abstinence were positively and linearly related to sperm concentration (slope: 3.74; p <0.01) and SDF (slope: 0.65; p = 0.044).

**Conclusions:**

According to our data, short ejaculatory abstinence is associated with better sperm quality. Indeed, a higher percentage of progressive sperm motility and lower levels of SDF have been reported in a short abstinence cohort. In contrast, the long abstinence group reported a higher sperm concentration.

**Systematic review registration:**

https://www.crd.york.ac.uk/PROSPERO/, identifier CRD42023472124.

## 1 Introduction

Despite variable and conflicting evidence on the decrease in male reproductive indices over the past half-century, male subfertility remains an area of concern with great academic, social, and financial interest worldwide (1). Apart from the extensively researched and conventionally accepted morphological, physiological, and genetic explanations of male infertility, the theory that the spermatocyte is a “cell in crisis” because its genetic material is under danger from multiple sources is also a current theory (2). In light of these theories and available data, recent studies have sought to gain a deeper understanding of the underlying mechanisms of oxidative stress, which is the main cause of sperm DNA fragmentation (SDF). However, it is unclear whether routine use of SDF is beneficial in certain populations, such as those with recurrent miscarriages, modifiable risk lifestyle factors, and infertility (3). Resolving practicalities concerning the standardization of sperm collection may also help SDF clarity and maximize its utility. Furthermore, improved sperm quality could result from improved standardization and specification of the ideal time for sperm collection (4). The physiology of ejaculation and the individual quantitative and qualitative contributions of seminal vesicles, prostate gland, and epididymis after repeated ejaculations, and the duration of ejaculation abstinence (EA) should be considered, as abstinence may affect conventional sperm parameters such as volume and total sperm count in both men with normospermia and dyspermia (5). This has also been reported to be the case with SDF, as the length of abstinence was positively correlated with semen volume, sperm concentration, and total sperm count, while SDF was significantly lower in shorter EA compared to the recommended (3–7 days) in healthy donors (6). In real-world applications, these observations seem to be of clinical importance, as standardization of sperm collection may improve some of the primary fertility endpoints. Borges et al. reported that EA of four days or less was associated to lower SDF, higher rates of fertilization and pregnancy comparing to longer ejaculatory abstinence in couples undergoing assisted reproductive technology (ART) (7). Despite accumulating evidence, the World Health Organization recommends a minimum of two days and a maximum of seven days of abstinence (8), a wide range that should be considered when interpreting sperm quality. In this systematic review and meta-analysis, we aimed to evaluate the association between ejaculation abstinence time and sperm quality in adult men undergoing a male infertility work-up from randomized clinical studies. By specifically focusing on RCTs, this study aims to report a higher level of evidence compared to observational or non-randomized studies.

## 2 Methods

### 2.1 Systematic literature search

This systematic review was conducted according to the Preferred Reporting Items for Systematic Reviews and Meta-analysis guidelines (PRISMA) (9) and was registered on PROSPERO (CRD42023472124), with the aim to answer the clinical question of whether ejaculatory abstinence affects sperm volume, sperm concentration, total motility, progressive motility, morphology, and SDF. In November 2022, we performed a search of major databases, and we collected data on adult men undergoing male infertility evaluation due to couple infertility or healthy donors. We performed a search in PubMed, Scopus, and Embase using the text terms ((“sperm parameters” OR “sperm analysis” [Mesh]) AND (“sperm DNA fragmentation” OR “DNA fragmentation” [Mesh]) AND (“sexual abstinence” [Mesh] OR “abstinence”)) and an advanced search in Scopus using the terms (“semen parameters” OR “sperm parameters” OR “DNA fragmentation”) AND (“abstinence”). Finally, we employed the snowball method to search for articles that were not identified in the first search. No time limitations were imposed on this study. We included studies reporting 1) the correlation between EA and sperm DNA fragmentation, and 2) comparisons of SDF or sperm parameters across various intervals of EA in both independent and paired groups. Studies involving non-adults, animals and retracted publications were excluded.

### 2.2 Study selection

A total of 279, 1,000, and 203 articles were selected from PubMed, Scopus, and Embase, respectively (**Supplementary Figure 1**). The citation lists of selected studies were manually checked, and references reported in the included articles were screened to find more potentially pertinent papers. After duplicates and only-abstracts have been removed, the authors assessed the eligibility and final inclusion in the meta-analysis of 13 studies. Studies were reviewed by two independent reviewers (ALG and GT); differences in opinion were discussed in consultation with the last author (GR), who solved discrepancies for further inclusion between the first two investigators. Design studies were also included.

### 2.3 Risk of bias assessment

Before the extraction of the outcomes, two reviewers (MGA and ALG) assessed the risk of bias concerning the following characteristics: random sequence generation (selection bias), allocation concealment (selection bias), blinding of outcome assessment (detection bias), incomplete outcome data (attrition bias), and selective reporting (reporting bias). To assess the risk of bias, the Cochrane risk of bias tool was used; a third reviewer (GIR) resolved disagreements between the reviewers’ judgements.

### 2.4 Data extraction

The authors of the study defined a two-day cut-off as a “short” or “long” abstinence period. All samples collected for <2 days (2 h to 2 days) were classified as short abstinence periods, while all samples collected from day 3 upward were considered as long abstinence periods. This period was arbitrarily defined based on the populations included and compared in the studies selected for meta-analysis. For each study, the difference between the long and short abstinence intervals was calculated to obtain the days of abstinence. In cases in which different interval times were reported in the same study, data were separately extracted and included in the forest plot.

### 2.5 Statistical analysis

Sperm parameters including semen volume, sperm concentration, total motility, progressive motility, SDF, and morphology were reported as mean ± SD. A meta-regression analysis has been performed among the mean difference between long and short abstinence in populations; the Galbraith plot was used to identify potential outliers among the populations included; when potential outliers were identified, a leave-one-out meta-analysis was subsequently performed. Finally, to identify a linear relationship between the days of abstinence and the parameter under consideration, a linear regression was performed between each sperm parameter and withdrawal time (reported as days between), and the outcome was reported with a bubble plot. All analyses have been performed with the software Stata/SE 17.0 for Mac (Apple Silicon), StataCorp 4905 Lakeway Dr College Station, TX 77845 USA. All reported p-values were based on two-sided tests and compared to a significance level of 5%.

## 3 Results

### 3.1 Characteristics of the study

After removing 240 duplicates, 1,265 records were screened, and 34 full-text articles were assessed for eligibility. Finally, 13 studies were included in the quantitative and qualitative analyses (6, 7, 10–20) (**Supplementary Figure 1**). The baseline characteristics of the included studies are listed in **Supplementary Table 1**. All studies included in the meta-analysis were RCT enrolling patients from infertility check-up visits or healthy volunteers. The final number of patients enrolled in the study (N) was 2315, ranging from 6 to 836.

### 3.2 Analysis

**Figure 1:**
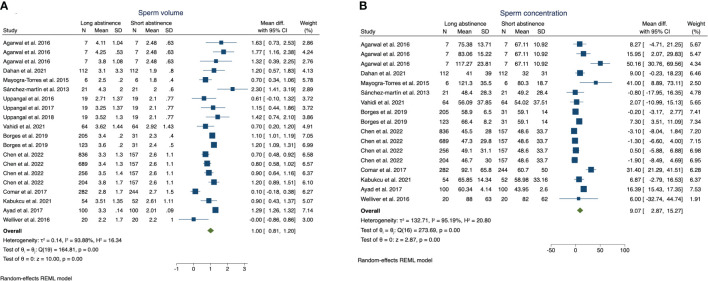
The forest plot of the sperm volume **(A)** and sperm concentration **(B)** changes according to long or short abstinence.

**Figure 2:**
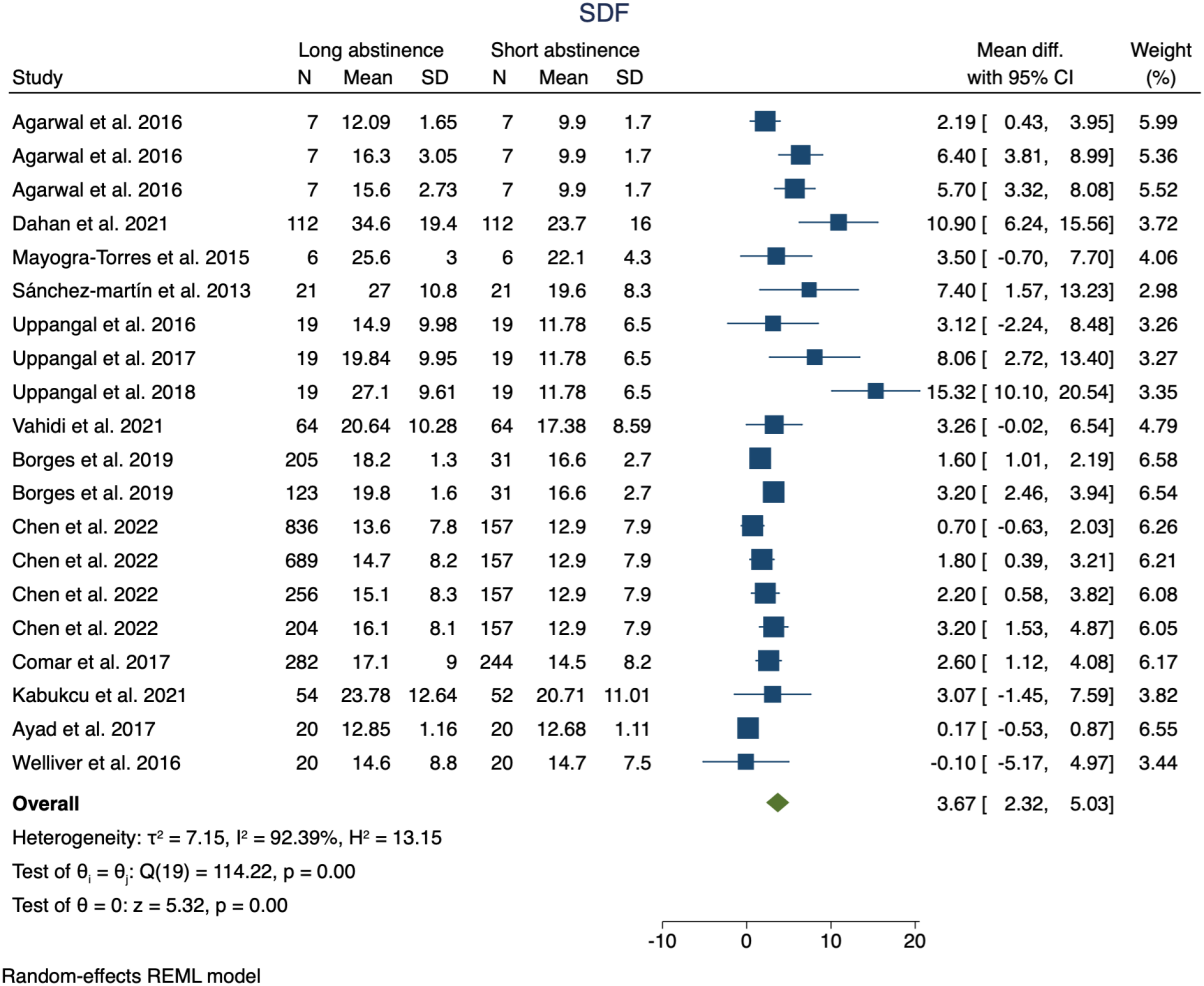
The forest plot shows of the sperm DNA fragmentation changes according to long or short abstinence.

We observed greater sperm volume (MD: 1; 95%CI 0.81–1.2; p <0.01) (**Figure 1**), sperm concentration (MD: 9.07; 95%CI 2.87–15.27; p <0.01) (**Figure 1**), and SDF (MD: 3.67; 95%CI 2.32–5.03; p <0.01) (**Figure 2**) in the long abstinence group than in the short abstinence group.

**Figure 3:**
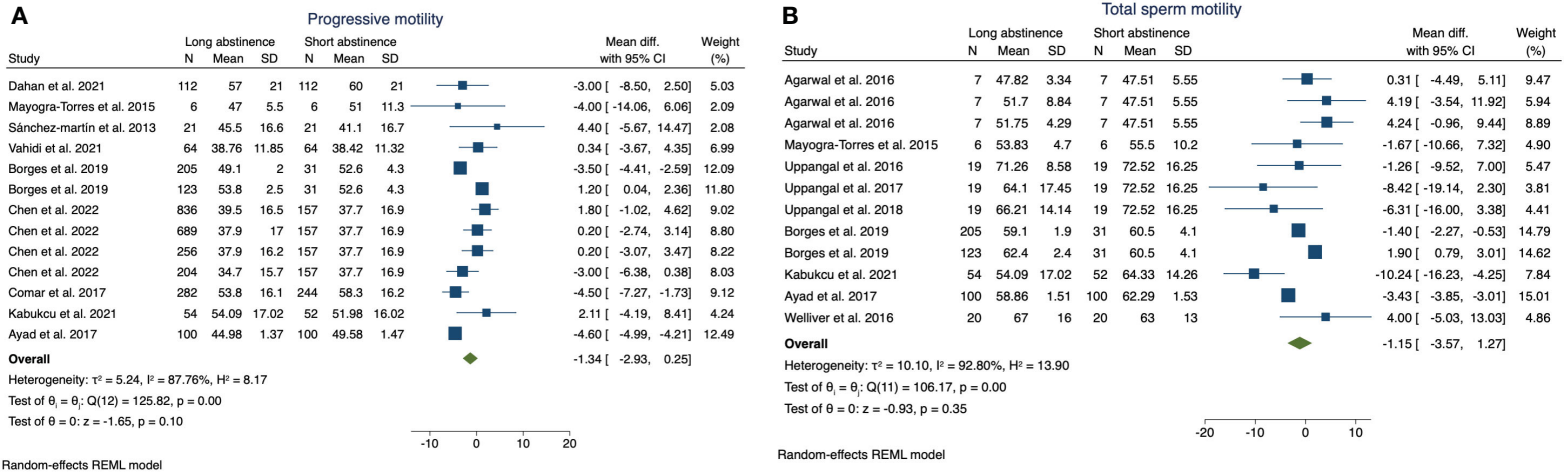
The forest plot shows of the sperm progressive motility **(A)** and total sperm motility **(B)** changes according to long or short abstinence.

Moreover, we observed a non-significant reduction in sperm progressive motility (MD: −1.34; p = 0.1) (**Figure 3A**) and total sperm motility (MD: −1.15; p = 0.35) in patients with longer vs. shorter abstinence times (**Figure 3B**).

**Figure 4:**
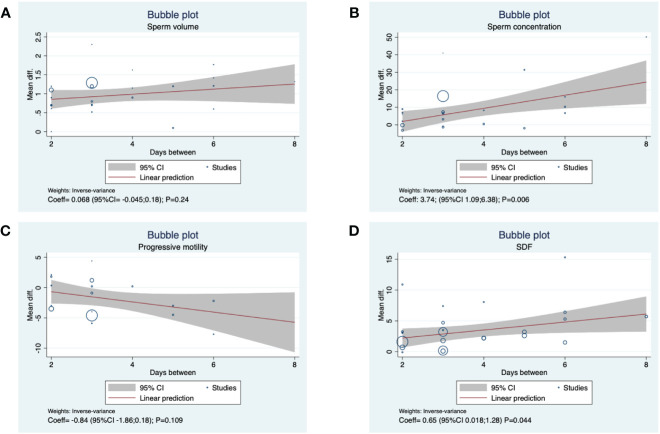
Meta regression analysis between days of abstinence and **(A)** sperm volume, **(B)** sperm concentration, **(C)** progressive motility, **(D)** SDF.

The meta-regression analysis revealed a positive association between days of abstinence and sperm concentration (slope: 3.74; 95%CI 1.09–6.38; p <0.01) and SDF (slope: 0.65; 95%CI 0.018–1.82; p = 0.044); otherwise, the meta-regression analysis did not highlight a statistically significant relationship between days of abstinence and volume (p = 0.24) and progressive sperm motility (p = 0.11) (**Figure 4**). **Supplementary Figure 2** shows the risk of bias of the included studies.

We used the Galbraith plot to test for heterogeneity among the included studies and identified potential outliers (**Supplementary Figure 3**). We performed a leave-one-out meta-analysis of sperm concentration (**Supplementary Figure 4**), sperm volume (**Supplementary Figure 5**), and SDF (**Supplementary Figure 6**). No changes in the statistical significance of the analysis were observed when the study was omitted.

## 4 Discussion

In the present meta-analysis, we showed that the number of days of ejaculatory abstinence significantly influenced sperm quality. Moreover, a statistically significant association was observed between longer ejaculatory abstinence and sperm volume, sperm concentration, and SDF has been showed. However, no relevant association between days of abstinence and total sperm motility was observed.

Overall, according to WHO criteria, a 2–7-day period of abstinence is recommended before collecting ejaculates for appropriate semen analysis (21).

However, the ideal period of ejaculatory abstinence remains debatable. Indeed, the recommendable period of abstinence worldwide is quite variable, and it is not well known how it could affect the final sperm analysis results.

Short abstinence periods (1–2 days) may result in higher sperm motility and viability due to reduced sperm aging and decreased sperm DNA damage (22). On the other hand, longer abstinence periods (5–7 days) may lead to higher sperm concentrations but lower motility and viability due to increased sperm senescence and oxidative stress (23). Therefore, a balance between abstinence duration and sperm quality is crucial for optimizing fertility outcomes in patients with infertility.

Short abstinence periods may reduce oxidative stress levels and improve antioxidant capacity by minimizing sperm exposure to reactive oxygen species (ROS) and lipid peroxidation. Conversely, longer abstinence periods may exacerbate oxidative stress and deplete antioxidant reserves, leading to sperm membrane damage and impaired fertility (24).

When sample collection takes place after a longer ejaculatory abstinence, the spermatozoa remain inside the epididymis for several days. This leads to alterations that are reflected in semen analysis results (11). Moreover, it is important to emphasize that individual factors may influence sperm parameters. Lifestyle habits and anthropometric parameters can affect sperm analysis (10). An interesting study conducted by Dahan et al. observed a relevant improvement in SDF when comparing the short abstinence group (3 h) and the long abstinence group (3 days). Authors registered an improvement of 30% or more in SDF parameters in the second sperm sample compared to the first one in 58 out of 112 patients. Moreover, the short abstinence group reported lower sperm volume and total sperm concentration, with augmented progressive sperm motility (14).

Because abstinence time is fundamental to ensure both the quality and quantity of spermatozoa required to achieve natural and assisted pregnancy, recent studies have focused on the achievement of a perfect sample (25). Due to the increasing interest in artificial insemination, research has suggested that shorter abstinence may be more appropriate in ART than the conventional abstinence recommended for routine semen analysis (11).

An interesting meta-analysis presented by Calogero et al. reported a very short abstinence period, especially among patients affected by OAT. After a brief period of abstinence (4 h), the authors reported improved sperm parameters in OAT-affected patients, including increased sperm concentration, total and progressive sperm motility, and decreased SDF levels (26).

However, a previous meta-analysis conducted on non-randomized clinical studies reported that short-term abstinence may be associated with limited improvements in semen quality in healthy men but could be more beneficial for infertile men, especially within the first 4 days of abstinence (27).

The positive association between days of abstinence and sperm concentration was a common finding in all papers analyzed. This relationship may be ascribed to the presence of stored sperm in the epididymis; therefore, a depleted sperm reserve and the consequent lower total sperm count in the shorter abstinence groups are predictable (28). The epididymis plays a fundamental role in sperm maturation, concentration, and survival. Moreover, during epididymal transit, spermatozoa acquire antioxidant enzymes (29).

Indeed, increasing evidence suggests that total antioxidant capacity (TAC) is considerably improved after reduced ejaculatory abstinence and a possible link between short abstinence, TAC, and SDF has been suggested (30).

**Figure 5:**
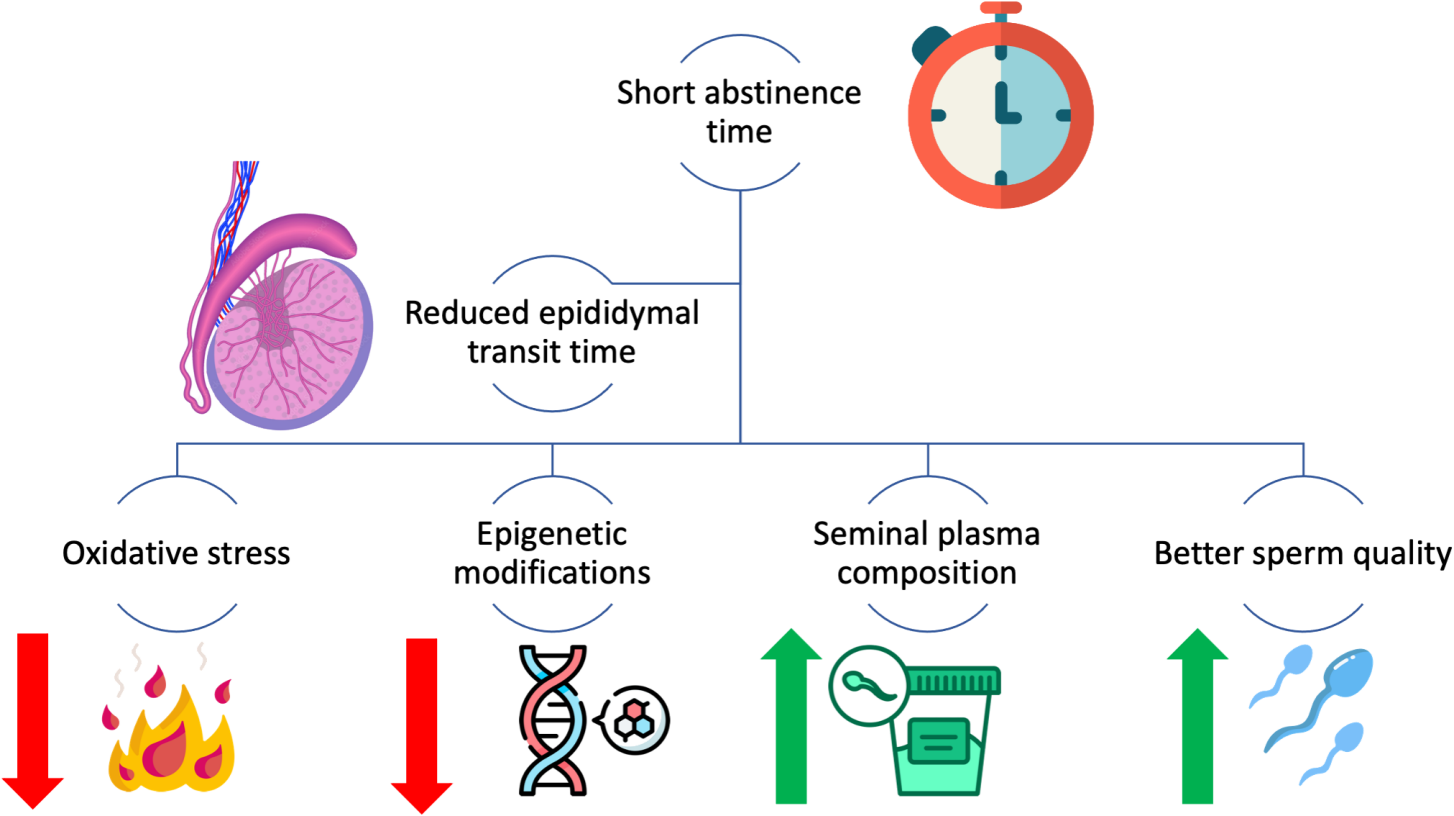
The impact of identified mechanisms influencing sperm quality in short abstinence time.

On the other hand, longer ejaculatory abstinence may lead to sperm functional alterations that may not be recognized by conventional sperm analysis, which may explain the lower pregnancy and fertility rate in long abstinence, notwithstanding the higher total sperm count and sperm concentration (7). **Figure 5** shows the impact of the identified mechanisms on sperm quality in the short abstinence time.

The strength of our study was that it explored and compared the effects of both long and short ejaculatory abstinence periods on various sperm parameters. By specifically focusing on RCTs, this study ensured a higher level of evidence than observational studies or non-randomized trials. This comparative analysis provides valuable insights into the optimal abstinence duration for improving sperm quality, which may have implications for fertility treatment and family planning. Our study investigated the impact of abstinence in healthy volunteers.

Before concluding, we would like to highlight some limitations of this study. First, we did not assess the pregnancy rate since it was not reported in the studies. Second, other markers of seminal oxidative stress were not reported in the studies, and they may be influenced by the time of abstinence.

## 5 Conclusions

According to our data, short ejaculatory abstinence is associated with better sperm parameters. Indeed, a higher percentage of progressive sperm motility and lower levels of SDF were reported in a short abstinence cohort. Otherwise, long abstinence group reported higher sperm concentration.

These results should be considered especially when counseling patients about ART.

Instead of adopting a one-size-fits-all approach to abstinence duration, future practices may involve personalized recommendations based on individual sperm quality and characteristics. Advanced diagnostics and biomarkers could help assess sperm health, allowing clinicians to tailor the abstinence period to maximize the sperm quality for each donor.

Future approaches can also include artificial intelligence and machine learning algorithms that can be employed to analyze large datasets of sperm quality parameters and donor characteristics to identify patterns and optimize the timing of sperm donation relative to abstinence periods.

## Author contributions

AL: Writing – review & editing, Writing – original draft, Formal analysis. MA: Validation, Writing – review & editing. SC: Writing – review & editing. AC: Writing – review & editing. MF: Writing – review & editing. MC: Writing – review & editing. AA: Writing – review & editing. PC: Writing – review & editing. AM: Writing – review & editing. GT: Writing – review & editing. CM: Writing – review & editing. GR: Writing – review & editing, Writing – original draft, Validation, Methodology, Investigation, Formal analysis, Data curation, Conceptualization.
