# Supplementary figures and images for: Effects of long and short ejaculatory abstinence on sperm parameters: a meta-analysis of randomized-controlled trials
Source: Front Endocrinol (Lausanne). 2024 May 17;15:1373426. doi: 10.3389/fendo.2024.1373426 (PMC11140051; doi:10.3389/fendo.2024.1373426)

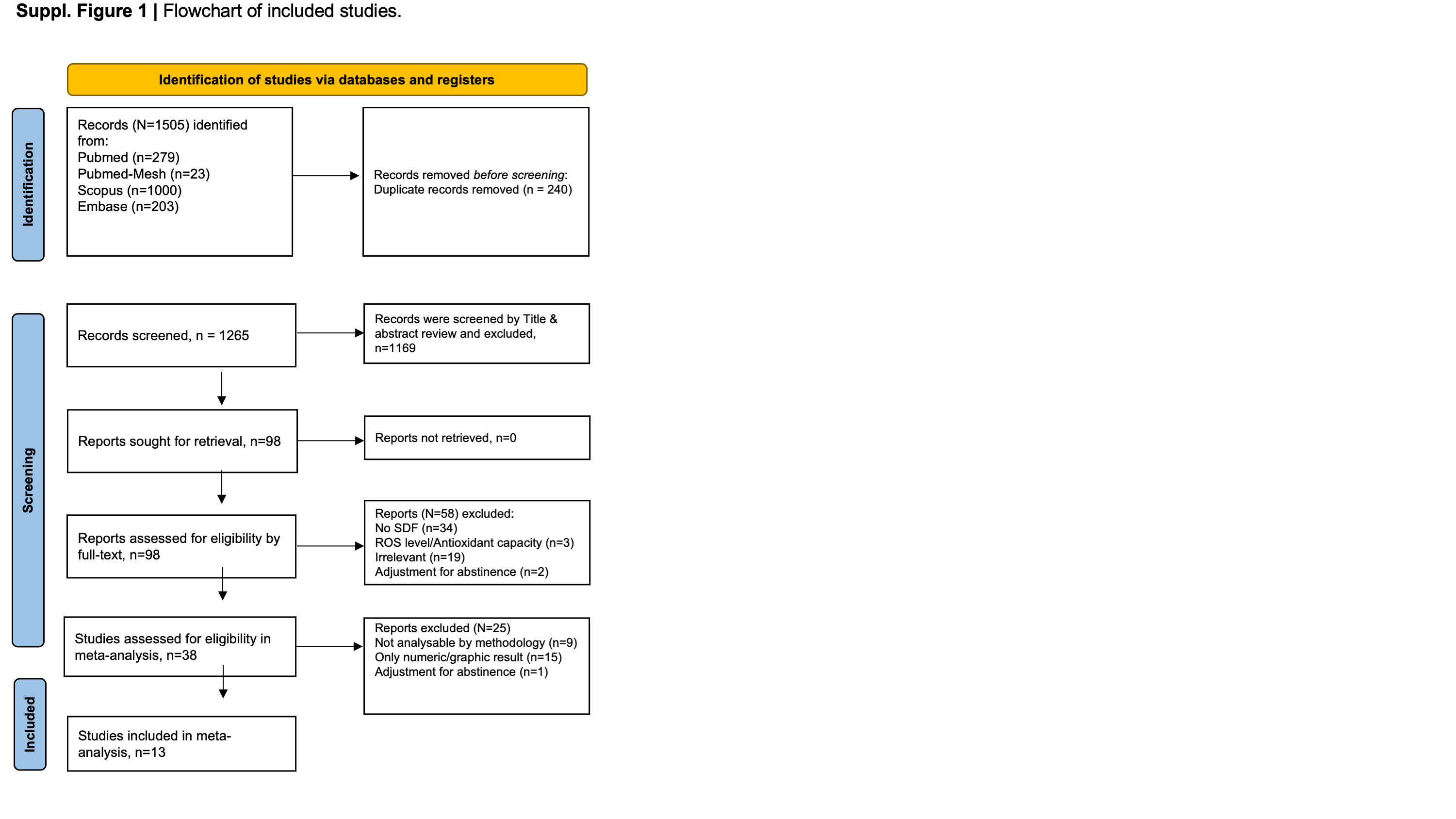

Supplement: Supplementary file 1 [file Image_1.tiff]

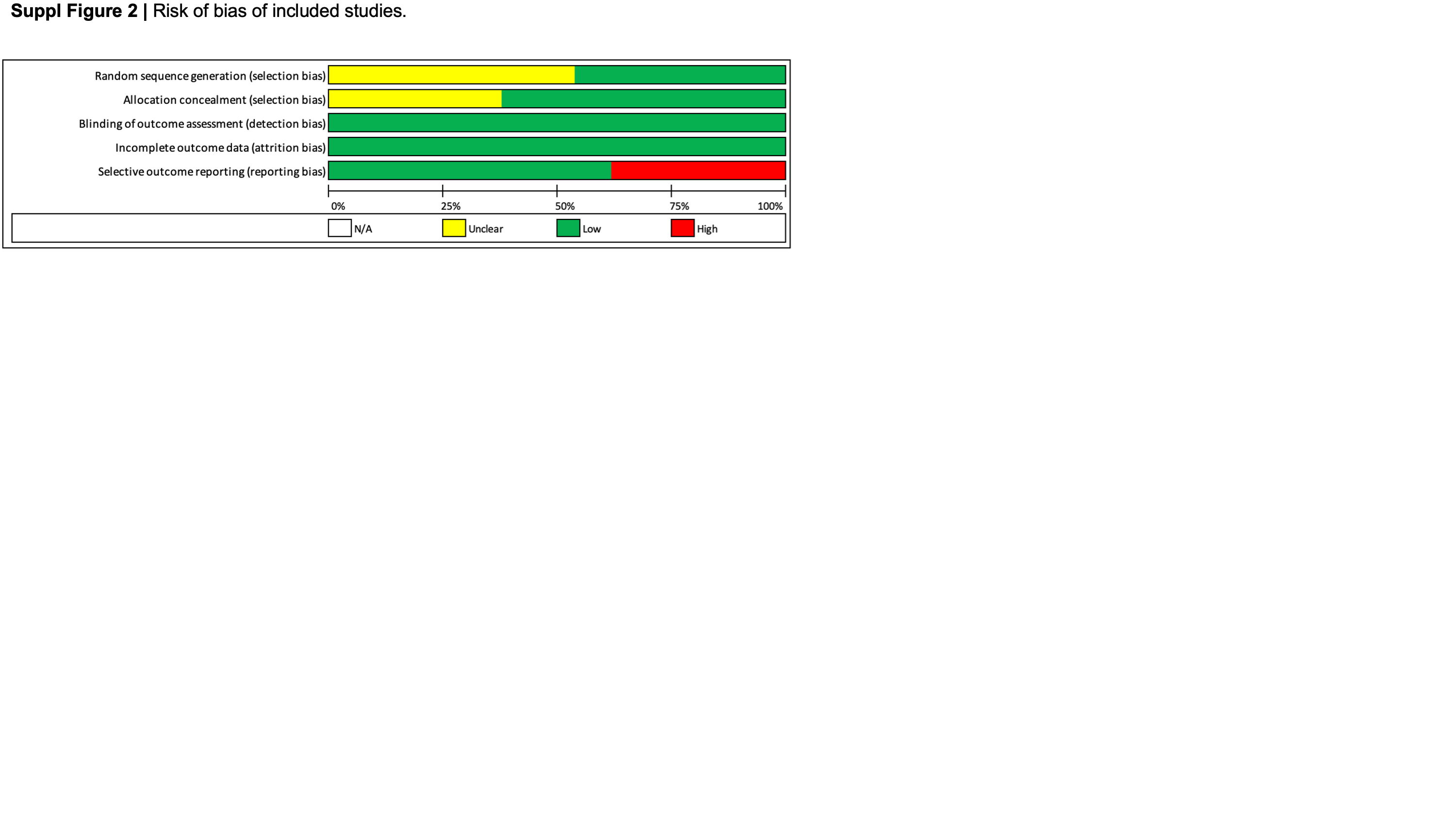

Supplement: Supplementary file 2 [file Image_2.tiff]

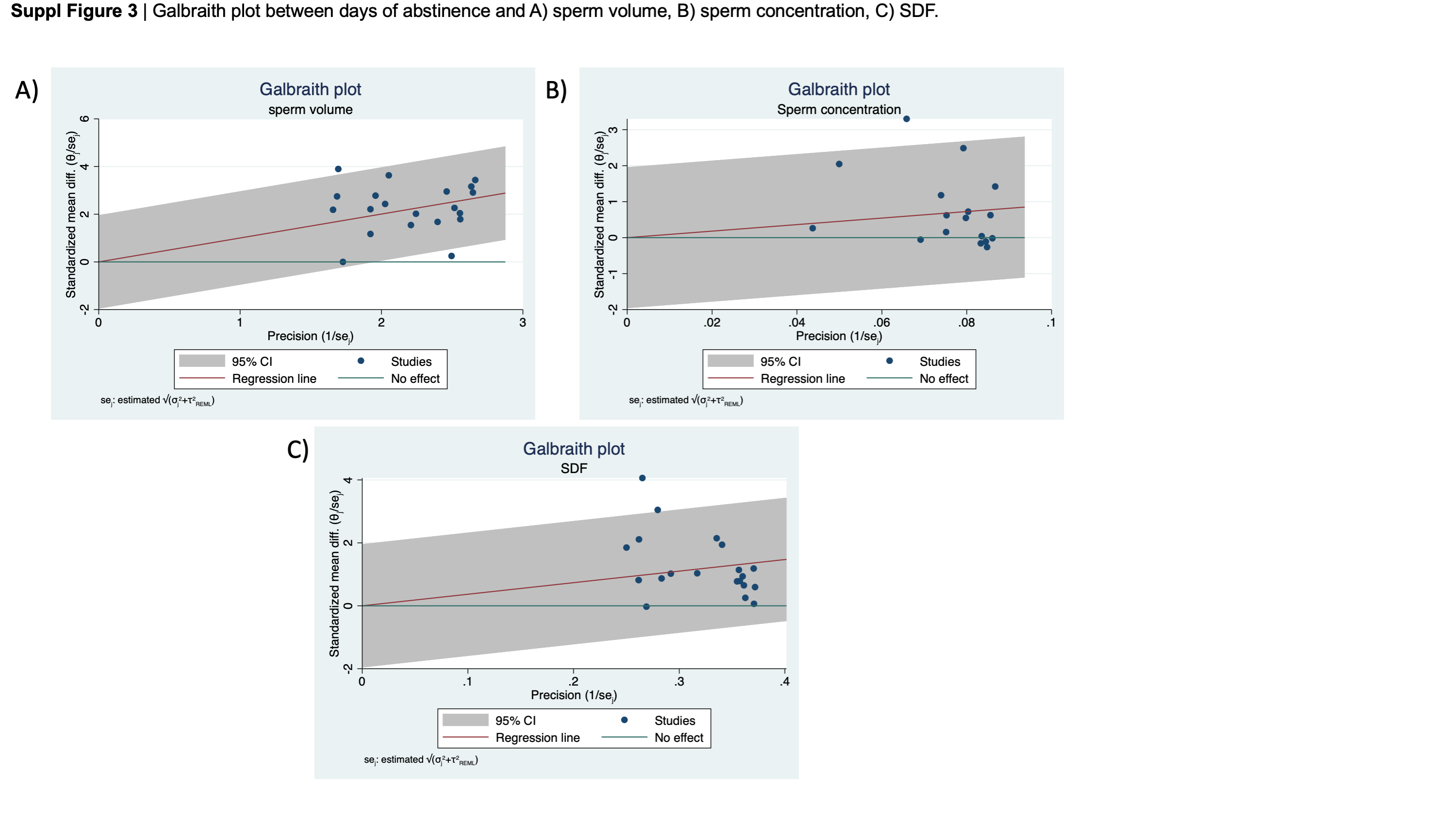

Supplement: Supplementary file 3 [file Image_3.tiff]

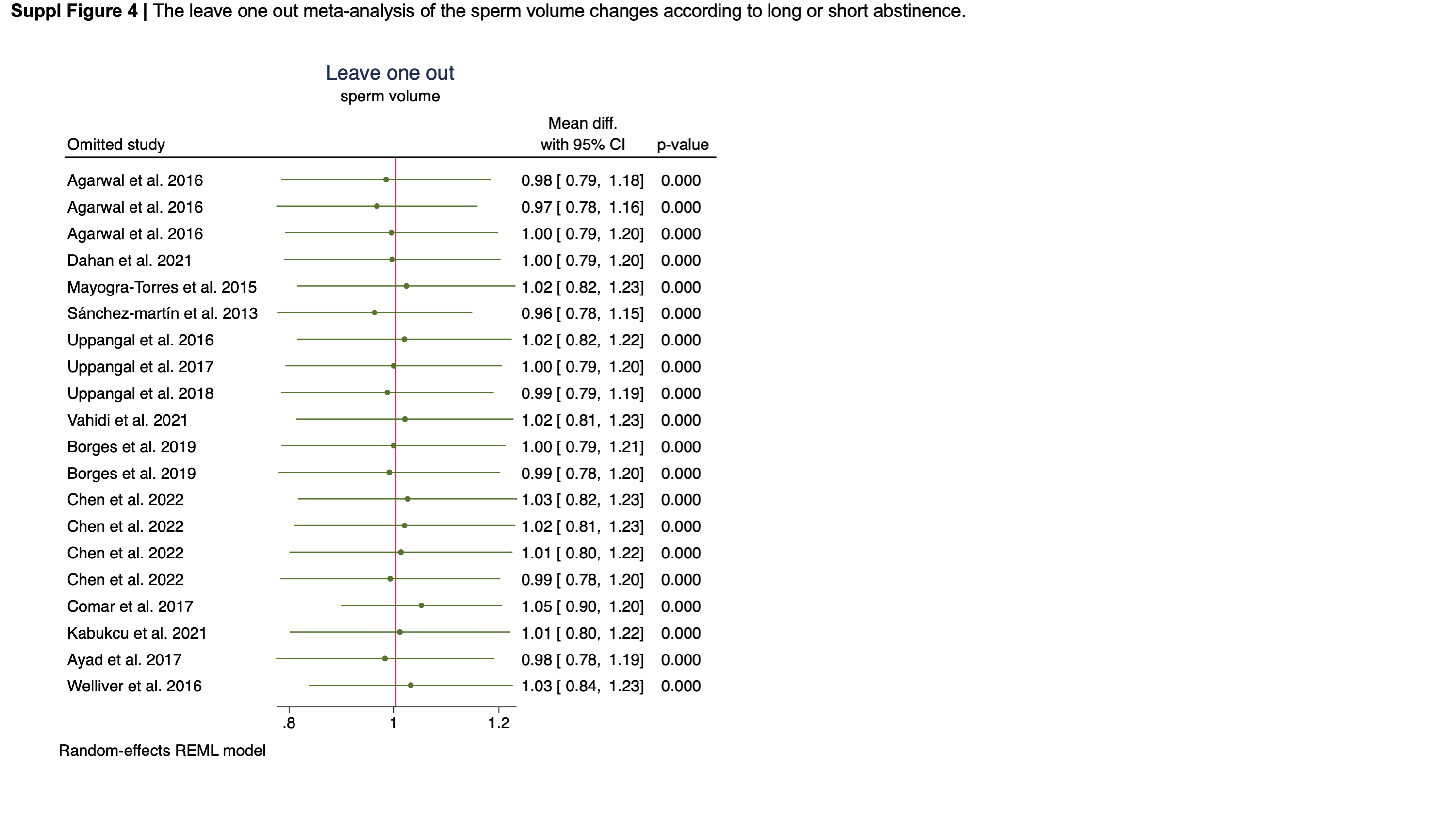

Supplement: Supplementary file 4 [file Image_4.tiff]

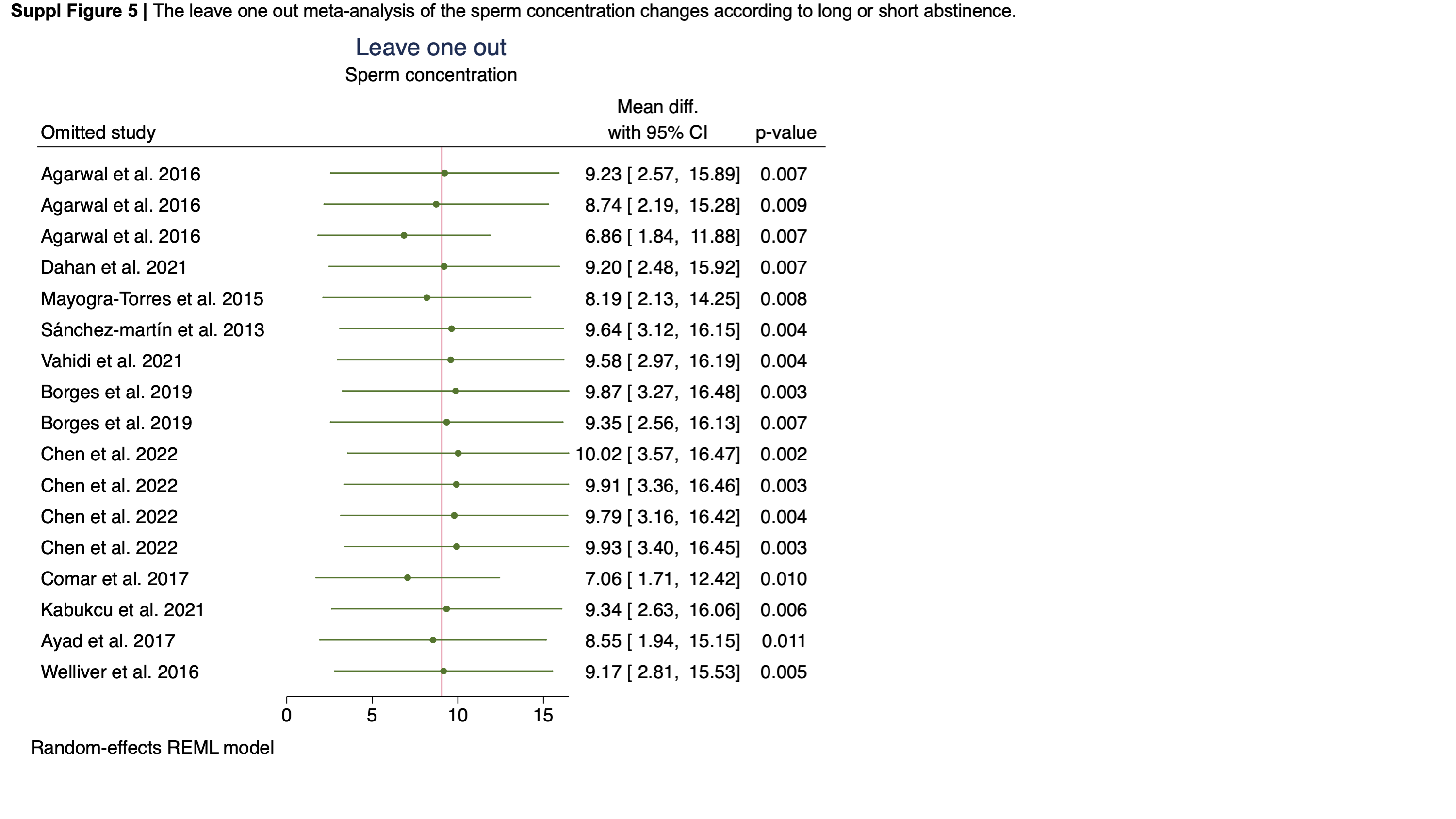

Supplement: Supplementary file 5 [file Image_5.tiff]

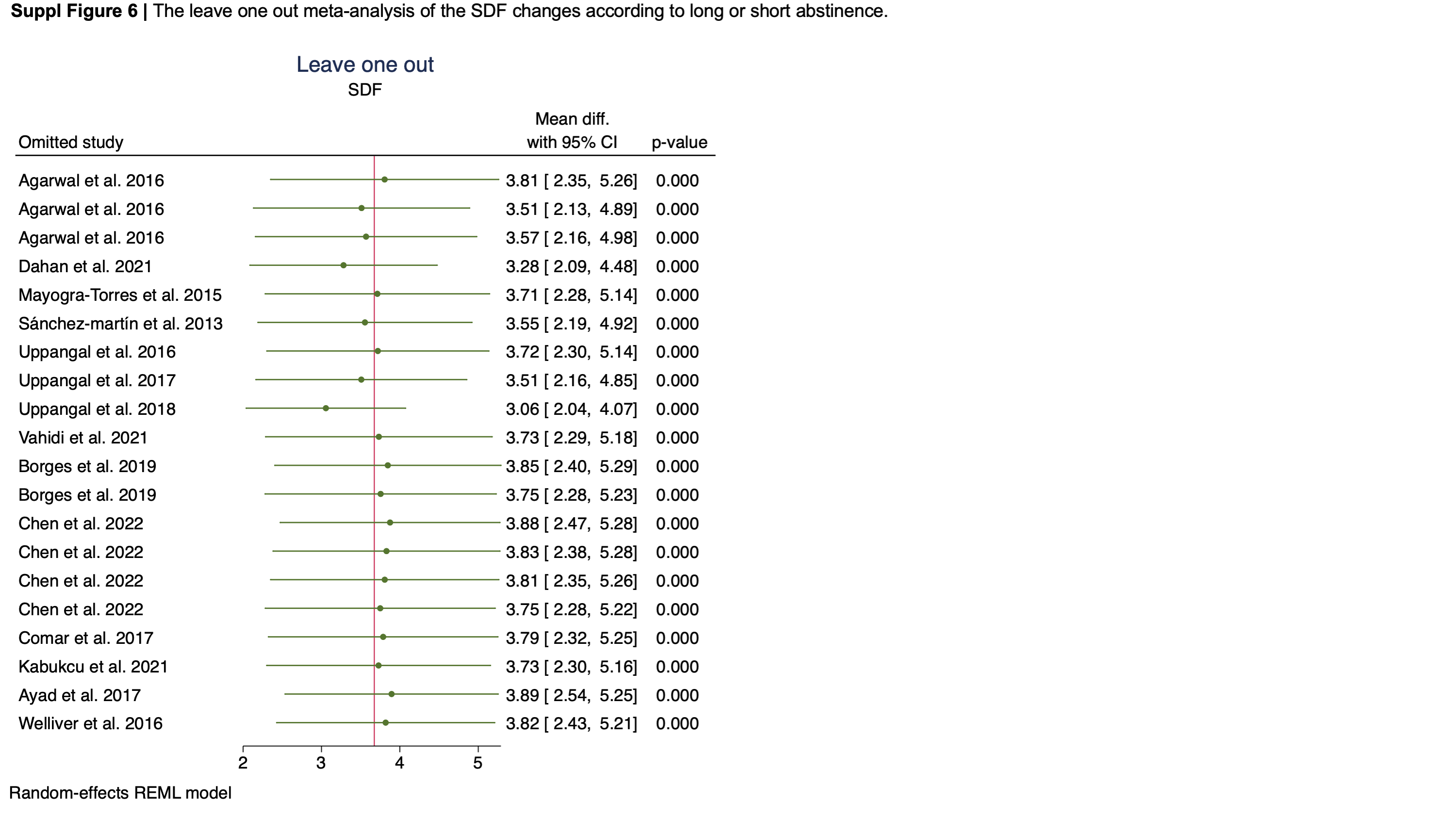

Supplement: Supplementary file 6 [file Image_6.tiff]
