# Supplementary material for: Effects of long and short ejaculatory abstinence on sperm parameters: a meta-analysis of randomized-controlled trials
Source: Front Endocrinol (Lausanne). 2024 May 17;15:1373426. doi: 10.3389/fendo.2024.1373426 (PMC11140051; doi:10.3389/fendo.2024.1373426)
Supplement: Supplementary file 7 [file Table_1.docx]

**Suppl. Table 1** | Characteristics of included studies

| **ID** | **Study name** | **Year** | **Population** | **Patients included** | **Day short** | **Day long** |
| --- | --- | --- | --- | --- | --- | --- |
| **1** | Agarwal et al | 2016 | Healthy volunteers | 14 | 1 | 5, 7, 9 |
| **2** | Dahan et al | 2021 | Subfertile men | 224 | 1 | 3 |
| **3** | Mayogra-Torres et al. | 2015 | Healthy volunteers | 12 | 1 | 4 |
| **4** | Sánchez-martín et al. | 2013 | Infertile and healthy volunteers | 42 | 1 | 4 |
| **5** | Uppangal et al. | 2016 | Healthy volunteers | 38 | 1 | 3, 5, 7 |
| **6** | Vahidi et al. | 2021 | Subfertile men | 128 | 1 | 3 |
| **7** | Borges et al. | 2019 | Random male population | 236 | 1 | 3, 4 |
| **8** | Chen et al. | 2022 | Random male population | 993 | 2 | 4, 5, 6, 7 |
| **9** | Comar et al. | 2017 | Random male population | 526 | 1 | 6 |
| **10** | Kabukcu et al. | 2021 | Infertile couple | 106 | 1 | 3 |
| **11** | Meitei et al. | 2022 | Normospermic and OAT | 421 | 1 | 4, 7 |
| **12** | Ayad et al. | 2017 | Healthy volunteers | 200 | <1 (4h) | 4 |
| **13** | Welliver et al. | 2016 | Healthy volunteers | 40 | 2 | 4 |
